# Effect of Electrical Conductivity Through the Bulk Doping of the Product of Titanocene Dichloride and 2-Nitro-1,4-phenylenediamine

**DOI:** 10.3390/jfb2010018

**Published:** 2011-03-17

**Authors:** Charles E. Carraher, Amitabh J. Battin, Michael R. Roner

**Affiliations:** 1 Department of Chemistry and Biochemistry, Florida Atlantic University, Boca Raton, FL 33431, USA; E-Mail: amitabhbattin@gmail.com; 2 Department of Biology, University of Texas at Arlington, Arlington, TX 76019, USA; E-Mail: roner@uta.edu

**Keywords:** metal-containing polymers, titanocene-containing polymers, conductivity, iodine doping, bulk conductivity, electrical conductivity

## Abstract

The condensation polymer derived from reaction between titanocene dichloride and 2-nitro-1,4-phenylenediamine was doped by mixing the polymer with different amounts of iodine. This bulk doping of the titanocene polyamine resulted in an increase in bulk conductivity from 10 to over 1,000 fold. Conductivity increased to a doping level of about 10 to 15% iodine. Conductivity decreased as the sample discs were heated returning to pre-doped levels after the samples were heated for eight minutes. It is believed that this decrease in conductivity is due to the surface evaporation of iodine as the samples were heated. MALDI MS and IR results are consistent with the formation of C-I compounds for doped materials.

## 1. Introduction

The search for non-metal electrically conductive materials has been ongoing for over 30 years. The importance of this effort was recognized by the awarding of the Nobel Prize in 2000 to MacDiarmid, Heeger, and Shirakawa for their work with polyacetylene [1,2,3,4,5,6]. Conductivity is dependent on the micro- or fine structure of the fibrils, doping agent, extent, technique, and aging of the sample as well as other factors so it is complex [7,8,9,10,11,12,13].

The transformation of materials from being semiconductors to conductors occurs through exposure of the polymeric materials to dopants that allow the materials to increase their conductivity ten- to 10^9^-fold. The structural requirement for successful doping is that the polymer chains possess what is referred to in molecular orbital terms as whole chain delocalization and in valence bond theory as whole or entire chain resonance such that the polymer chain acts as a conduit for electrical charge to transverse along the chain.

We have been involved in the search for semiconductors that contain metals within coordination and condensation polymers for about 30 years focusing on polymer chains that can exhibit whole chain resonance with many of these being semiconductors [14,15,16,17,18,19,20,21,22,23].

**Figure 1:**
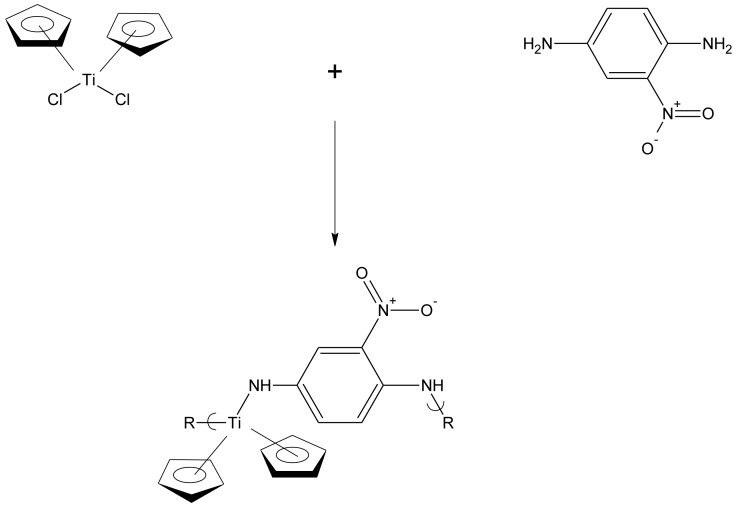
Synthesis of polymer from the reaction of titanocene dichloride and 2-nitro-1,4-phenylenediamine where R is simple chain extension.

Recently we began doping some of these polymers. One of these polymers was derived from the condensation reaction of titanocene dichloride and 2-nitro-1,4-diamine (Figure 1). We recently reported on the successful doping of this product where surface exposure to iodine vapors resulted in the increase in conductivity to 10^4^ [24]. This was the initial report of a condensation polymer being successfully doped to significantly increase its conductivity. The current paper contains the initial report where simple bulk doping of a condensation polymer has significantly increased the polymer's conductivity.

We originally reported the synthesis of titanocene-containing polymers in 1971 [25]. The topic of metallocene-containing polymers was recently reviewed [26]. Some of these polymers were found to spontaneously form fibers [27], were able to moderate laser emission when present in samples at levels of ppm [30], and act as plant growth hormone carriers [28,29]. We have also found that some of these polymers are potent anticancer agents [31,32]. In 1972 we reported the synthesis of titanocene polyamines of the form studied in the present investigation employing the solution and interfacial techniques [33,34].

Here we report on our initial efforts aimed at increasing the electrical conductivity of these materials using bulk doping with iodine.

## 2. Results and Discussion

### 2.1. General

**Figure 2:**
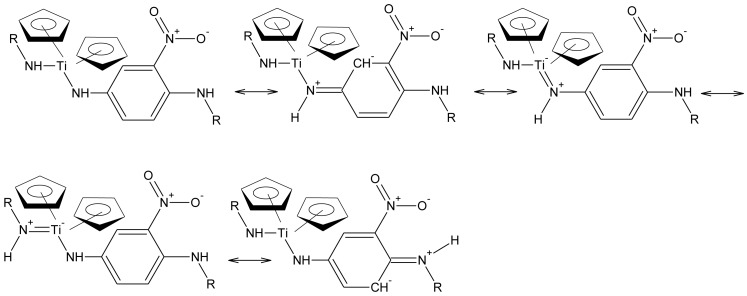
Resonance structures for 2-nitro-1,4-phenylenediamine unit where R = chain extension.

We have just begun investigating the results of polymer doping. While there are a number of different doping procedures employed in the production of conducting polymers, the most widely employed is the exposure of the polymeric material, often in a compacted disk, to elemental iodine vapors either as a vapor or as part of a mixture as we have done in the present study [1,7,8,9,10,11,35,36,37,38,39,40,41,42,43]. We studied the effects of doping of over 50 products. Of these, we found that one polymer did respond to a decent amount when doped with iodine vapors. The material was the product from the reaction between titanocene dichloride and 2-nitro-1,4-phenylenediamine (Figure 1). The titanium atom in titanocene dichloride has a vacant orbital that can accept electrons allowing resonance to occur through it [44]. The polymer can exhibit whole-chain resonance as shown in Figure 2 so its structure is consistent with the general structural criteria for conductivity. The product employed in the current study was synthesized in 45% yield employing the classical aqueous interfacial polycondensation process as described in the Experimental section. It has an average molecular weight of 2.4 × 10^4^ corresponding to a chain length (degree of polymerization) of 70 units.

### 2.2. Variation of Dissipation Factor and Dielectric Constant for Doped and Undoped Materials

Since the emphasis is on conductivity we will focus on this factor. Even so, it is of interest to briefly describe the various measured values that are employed in determining conductivity and to compare these values for the doped and undoped materials.

The dielectric constant, K, for the undoped samples varies from 0.4 to 1.9, indicative of materials with a relatively low dielectric constant. The dielectric constants for the doped products have increased varying from 8 to 38. For comparison, dielectric constants (60 HZ) for typical polymers vary from about 2 for polytetrafluoroethylene to 8.4 for poly(vinylidene fluoride) with values for nylons, polycarbonates, and polyesters generally in the range of 3 to 5 [12,13]. Values of K for the highly polar Group IVB polyoximes [17,22,23] varied from 2 to 10 and 5 to 120 for a series of palladium polyamines [14]. Both the polyoxime and polyamine samples tested can exhibit whole-chain resonance in their backbones.

The dissipation factor, D, varies from 0.4 to 1.4 for the undoped samples and from 0.4 to 2.7 for the doped samples. Thus, the dissipation factors do not widely vary when the sample is doped. Values for D for the Group IVB polyoximes [17,22,23] vary from 2.6 to 10 and for the palladium polyamines [14] from 0.2 to 28. Values for commercial polymers are as follows: nylon 66- 0.02; polyethylene- 0.0002; polypropylene- 0.0003; polytetrafluoroethylene- 0.0003; and poly(vinyl chloride)- 0.01 [12,13]. In general, more polar molecules have higher D values.

The D and K values are consistent with the major changes found between the doped and undoped samples being contained in the K term. The K term is largely a measure of the polarity of the material so that the larger values found for the doped samples are consistent with the materials becoming more polar as they are doped. This trend is found for all of the samples reported on here.

In general, changes in conductivity with applied frequency are usual and the particular trend varies with the particular material [12,13,35,40].

### 2.3. Effect of Amount of Iodine

**Figure 3:**
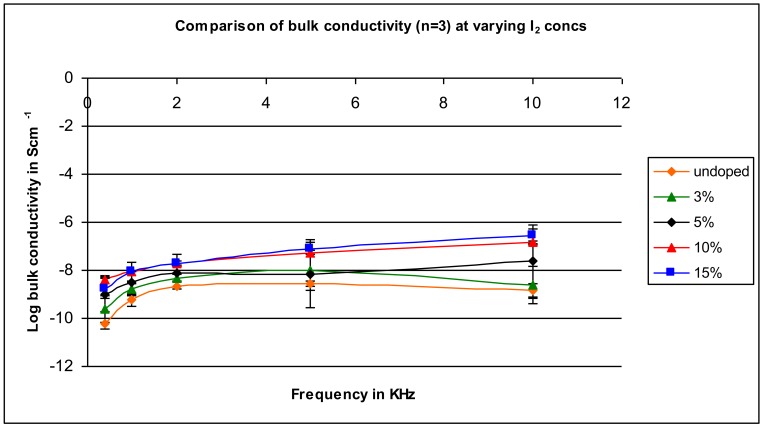
Plot of log bulk conductivity as a function of frequency for samples containing varying amounts of iodine from 0% to 15%.

In this study, specific amounts of iodine are mixed with the polymer. Figure 3 contains the conductivity for four amounts of iodine added, from 3% to 15%. Unlike doping silicon to produce semiconductors where the amount of doping agent is generally less than 1%, the amounts of iodine employed to move polyacetylene from a semiconductor to a conductor are in the range of 3 to 15% [1,2,3,4,5,6,7,35,40]. Thus, it was this range that was studied. Bulk conductivity generally increased as the amount of iodine increased, increasing generally over 100-fold for all frequencies for the 15% doped samples compared to the undoped sample. The greatest increase was found for the highest applied frequency with conductivity increasing about 1,000-fold converting the material from being a near non-conductor to being a near-conductor. The changes from addition of 10% compared to 15% iodine are small.

### 2.4. Effect of Heating

**Figure 4:**
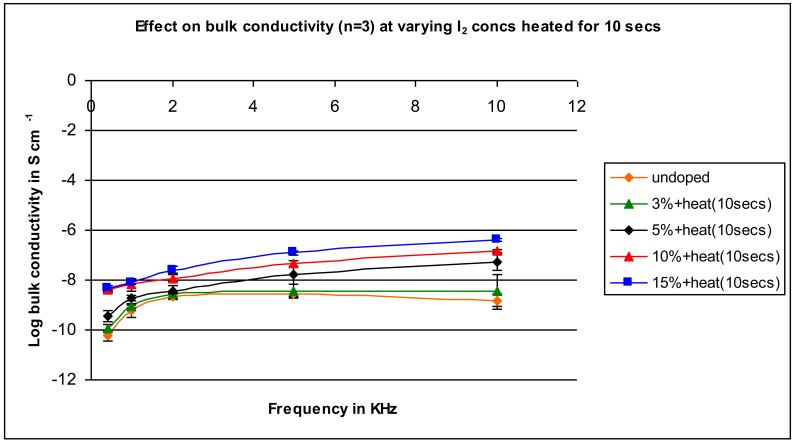
Conductivity of samples containing varying iodine amounts heated for 10 seconds as a function of applied frequency.

**Figure 5:**
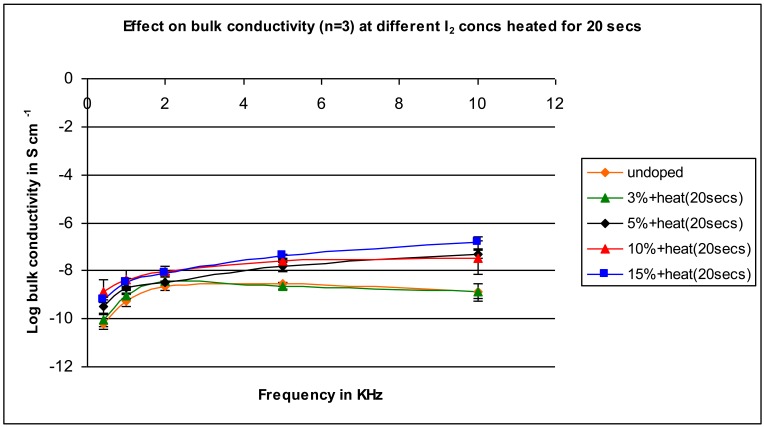
Conductivity for samples mixed with varying iodine amounts heated for 20 seconds as a function of applied frequency.

**Figure 6:**
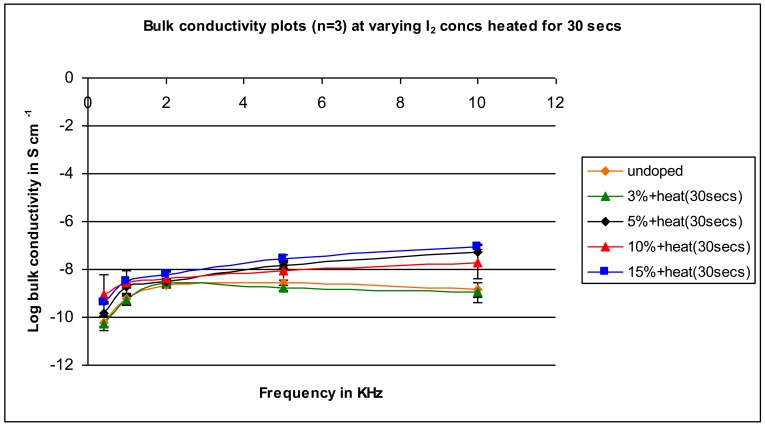
Conductivity for samples mixed with varying iodine amounts heated for 30 seconds as a function of applied frequency.

**Figure 7:**
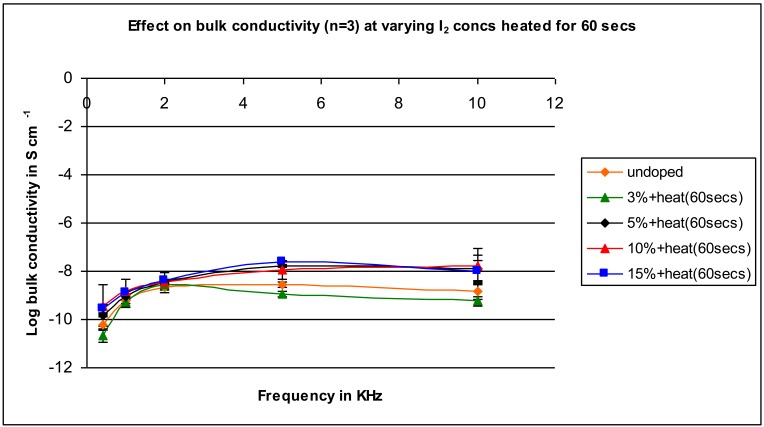
Conductivity for samples mixed with varying iodine amounts heated for 60 seconds as a function of applied frequency.

**Figure 8:**
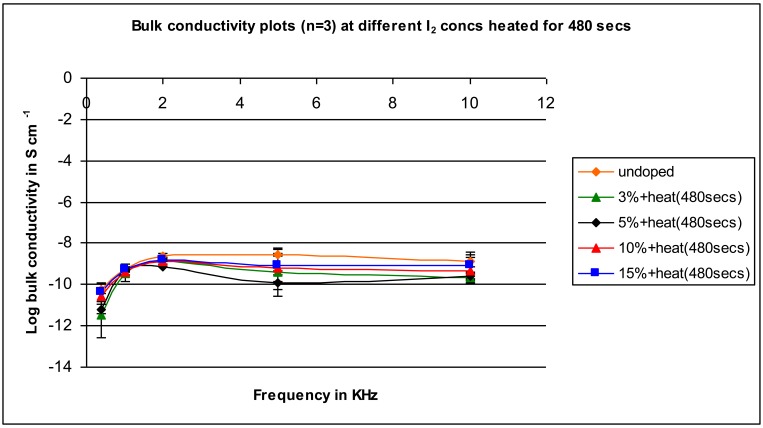
Conductivity for samples mixed with varying iodine amounts heated for 480 seconds as a function of applied frequency.

In the first study, the iodine was simply present as part of a mixture brought together with mixing. More effective mixing might occur when the sample is heated since the iodine is volatilized and should disperse within the sample. Further, heating may help in the formation of an active compound allowing for better conductivity. Figure 4, Figure 5, Figure 6, Figure 7 and 8 contain results for different heating times.

A similar pattern to that found for the unheated samples is found for the samples heated for 10 seconds (compare Figures 3 (unheated) and 4 (heated)). Conductivity increases as the concentration of iodine is increased with the difference between the conductivity of the undoped and doped in the range of 100- to 1,000-fold. The results are similar for the samples heated for 20 seconds (Figure 5) and 30 seconds heating (Figure 6).

For a heating time of 60 seconds (Figure 7) the conductivity difference between the doped and undoped samples is less, with differences being several fold to 10-fold with the conductivity of the 3% doped sample approximating that of the undoped sample.

After heating for 480 seconds (Figure 8), all of the samples approximate the conductivity of the undoped sample.

In general, as the heating times increase, the conductivity of the doped samples decrease eventually returning to the conductivity of the undoped sample. We believe that this is due to the heating preferentially removing surface iodine so that while more effective mixing may be occurring internally, it is not experimentally observed because of the removal of the surface iodine. As noted before, the measurements are made using two flat electrodes so that surface phenomena is critical to the overall measurement and conductivity may well be different within the bulk of the sample. The suggestion that the decrease is due to evaporation of surface iodine is consistent with a decrease in metallic coloring on the surfaces of the sample disks as heating is increased.

### 2.5. Physical Characterization

It is obvious that the doping of the material caused an increase in conductivity. We made several studies to investigate if differences between the doped and undoped materials could be found and identified.

Comparisons of the doped and undoped materials were made over the infrared range of 4,000 to 400 wave numbers (all band assignments are given in wave numbers). There were few differences. There was a modest difference in the Ti-N band occurring at 496 for the undoped polymer and 473 for the doped material. Further, there is a new band found at 664 for the doped sample tentatively assigned to the formation of the C-I moiety [45,46]. It is possible that activation occurs through the formation of the C-I moiety.

**Figure 9:**
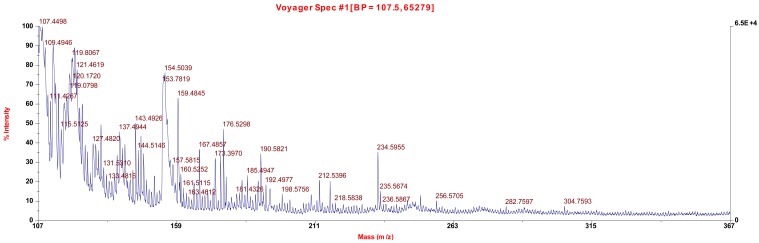
MALDI MS for the undoped material.

**Figure 10:**
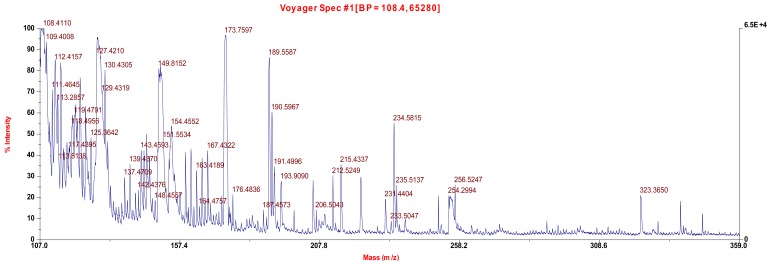
MALDI MS for doped material containing 10% iodine.

MALDI MS was also performed on the undoped and doped samples and the results compared (Figure 9 and Figure 10). Three new ion fragments were found for the doped material. The ion fragment at 254 Da is assigned to molecular iodine, I_2_. The ion fragment at 232 Da is assigned to HNPhNHI and the ion fragment at 323 is assigned to TiNHPhNO_2_NHI, both consistent with the formation of an aromatic C-I moiety.

It is unknown if the formation of the C-I moiety is the activating step necessary to initiate the increase in conductivity or if it is simply a consequence of the activating step.

## 3. Experimental Section

### 3.1. Synthesis

Titanocene dichloride (CAS # 1271-19-8) and 2-nitro-1,4-phenylenediamine (CAS # 5307-14-2) were used as received from Aldrich Chemical Company, Inc, Milwaukee, WI, USA.

2-Nitro-1,4-phenylenediamine (3.00 mmoL) was dissolved in 30 mL of water. Sodium hydroxide (6.0 mmoL) was also dissolved in the water. Titanocene dichloride (3.00 mmoL) was dissolved in 30 mL chloroform. The aqueous phase was first added to the Kimex emulsifying jar. Stirring (about 18,000 rpm) was begun and the chloroform phase was quickly added (3 to 4 seconds addition time) and stirring was continued for an additional 15 seconds. The dark brown precipitate was collected using suction filtration and washed with water and chloroform to remove unreacted materials. The solid was washed into a glass Petri dish and allowed to dry under room conditions of temperature and pressure.

### 3.2. Electrical Measurements

A standard procedure was employed to obtain the electrical measurements [1,7,8,9,10,11,35,36,37,38,39,40,41,42,43]. Briefly, a known amount of sample and iodine were mixed together with grinding to form a powder. The 2-nitro-1,4-phenylene diamine and titanocene dichloride polymer was finely ground to a powder employing a mortar and pestle in the usual fashion and viewed under an optical microscope (Olympus CH30 microscope) to determine an average size of the particles. The particles were irregular spheres with an average diameter of 1.02 × 10^−6^ (± 0.04) for (n = 20) meters. The powder was pressed into pellets using a Carver Laboratory Press. These pellets were utilized for the conductivity measurements. The thickness and diameter of the sample pellet were measured using a micrometer screw gauge. Pellet thickness (0.59 mm) and diameter (13.0 mm) were held constant for the current studies. A GenRad 1650 B impedance bridge was employed for the electrical measurements. The sample holder consists of an enclosed cage which houses the copper electrodes. Pellets were placed between the copper electrodes and an alternating current applied. The sample holder was connected to the impedance bridge which measures the various electrical properties. The impedance bridge was also connected to a 1311-A audio oscillator which allowed the frequency to be varied. The conductivity measurements were done for the following frequencies: 0.4 KHz, 1KHz, 2KHz, 5KHz and 10KHz. Measurements and calculations were made in the usual manner [1,7,8,9,10,^11^]. A blanket of nitrogen was present during the doping and measurement procedures to maintain an oxygen and moisture free surrounding. The assembly was calibrated employing materials with known conductivities.

Sample heating was carried out by placing the disks into a preheated oven set at 50 °C for the specified time.

Infrared spectra were obtained employing a JASCO FT/IR 4100. MALDI MS were obtained employing a high resolution electron impact positive ion matrix assisted laser desorption ionization time of flight, HR MALDI-TOF, mass spectrometry employing a Voyager-DE STR BioSpectrometer, Applied Biosystems, Foster City, CA, USA. The standard settings were used with a linear mode of operation and an accelerating voltage of 25,000 volts; grid voltage 90% and an acquisition mass range of 100–1,000 Da. Fifty to two hundred shots were typically taken for each spectrum. Molecular weight was determined employing a Brice-Phoenix Universal Light Scattering Photometer.

## 4. Conclusions

Several conclusions are evident from these studies. First, conductivity varies with the applied frequency. Second, conductivity generally decreases with increased heating times. Third, conductivity generally increases with the amount of iodine added to the sample with 10% and 15% additions offering generally similar conductivities. Fourth, the increases in conductivity are similar to the same magnitude found for surface doping [24]. Further, the variability of conductivity with respect to applied voltage is also similar to that found for the surface-doped samples [24]. Since most electrical measurements on polymers involve only surface measurements, the relationship between surface and bulk conductivities is not well established.

### 4.1. Future

We are currently investigating the extent of the structure-electronic behavior window. Conductivity increase is related to many factors including orientation of the polymer chains. For this study, the chains are probably preferentially oriented at right angles to the direction of measurement so that the true increases in conductivity may be much larger than those measured. This is a result of how the pellet is formed. The pellet is prepared by simply adding the (mixed) sample to a bore and pressure applied presumably orienting at least some of the chains at right angles to the applied pressure.

These polymers are rapidly synthesized employing a system that has been already employed industrially [12,13] utilizing commercially available reactants. Thus, they can be produced rapidly and easily.

These materials may offer some advantages to most other materials that must be doped to achieve the desired conductivity. While not reported here, the doped materials retain their conductivities even when left in the open air for several days. Thus, they appear to have good atmospheric stability. Because they have backbones that are different from most vinyl-intense polymers, they may offer a variety of different properties, some of which may be useful.

It would be interesting to see if other condensation polymers might show similar behavior. Of particular interest would be those that contain aromatic units throughout their backbone such as some of the aromatic nylons.

## References

[1] Chiang C.K., Druy M.A., Gau S.C., Heeger A.J., Louis E.J., MacDiarmid A.G., Park Y.W., Shirakawa H. (1978). Synthesis of Highly Conducting Films of Derivatives of Polyacetylene, (CH)x. J. Am. Chem. Soc..

[2] Kaner R.B., MacDiarmid A.G. (1988). Plastics That Conduct Electricity. Sci. Am..

[3] MacDiarmid A.G., Epstein A.J. (1991). “Synthetic Metals”: A Novel Role for Organic Polymers. Macromol. Chem..

[4] MacDiarmid A.G., Epstein A.J., Prasad P.N., Nigam J.K. (1991). Science and Technology of Conducting Polymers. Frontiers of Polymer Research.

[5] Hohnholz D., MacDiarmid A.G. (2001). Line Patterning of Conducting Polymers: New Horizons for Inexpensive, Disposable Electronic Devices. Synth. Met..

[6] MacDiarmid A.G. (2003). Twenty-five Years of Conducting Polymers. Chem. Comm..

[7] Jozefiak T.H., Ginsburg E.J., Gorman C.B., Grubbs R.H., Lewis N.S. (1993). Voltammetric Characterization of Soluble Polyacetylene Derivatives Obtained from the Ring-Opening Metathesis Polymerization (ROMP) of Substituted Cyclooctatetraenes. J. Am. Chem. Soc..

[8] Gorman C.B., Ginsburg E.J., Grubbs R.H. (1993). Soluble, Highly Conjugated Derivatives of Polyacetylene from the Ring-Opening Metathesis Polymerization of Monosubstituted Cyclooctatetraenes: Synthesis and the Relationship between Polymer Structure and Physical Properties. J. Am. Chem. Soc..

[9] Langsdorf B.L., Zhou X., Lonergan M.C. (2001). Kinetic Study of the Ring-Opening Metathesis Polymerization of Ionically Functionalized Cyclooctatetraenes. Macromolecules.

[10] MacDiarmid A.G. (2001). Nobel Lecture: Synthetic metals: A novel role for organic polymers. Revs. Mod. Phys..

[11] Shirakawa H. (2001). Nobel Lecture: The discovery of polyacetylene film-the drawing of an era of conducting polymers. Revs. Mod. Phys..

[12] Carraher C. (2011). Polymer Chemistry.

[13] Carraher C. (2010). Introduction to Polymer Chemistry.

[14] Carraher C., Nwufoch V., Taylor J.R. (1989). Electrical properties of palladium polyamines with respect to the theory of whole chain resonance. Polym. Mater. Sci. Eng..

[15] Carraher C., Venable W., Blaxall H., Sheats J. (1980). Synthesis and characterization of antimony V-polycobalticinium esters. J. Macromol. Sci. Chem..

[16] Carraher C., Blaxall H., Schroeder J., Venable W. (1978). Synthesis and Electrical, Thermal and Solution Characterization of Antimony V Polyesters. Org. Coat. Plast. Chem..

[17] Carraher C., Christensen M., Schroeder J. (1977). Physical characterization of titanium polyferrocene oximes. J. Macromol. Sci. Chem..

[18] Carraher C., Lanz L. (2005). Synthesis and physical characterization of group IVB metallocene polymers containing norfloxacin. J. Polym. Mater..

[19] Carraher C., Leahy D., Ailts S. (1977). Initial Electrical Conductivity Measurements of Selected Organometallic Polymers. Org. Coat. Plast. Chem..

[20] Carraher C., Schroeder J., Venable W., McNeely C. (1978). Synthesis and electrical, thermal and solution characterization of antimony V polyesters. Org. Coat. Plast. Chem..

[21] Carraher C., Schroeder J., Venable W., McNeely C., Giron D., Woelk W., Feddrson M. (1978). Electrical, Solvent, Thermal and Fungal Properties of Organotin-Containing Poly(ethyleneimine). Additives for Plastics.

[22] Carraher C., Manek T., Linville R., Taylor J.R., Torre L., Venable W. (1980). Preliminary AC and DC Electrical Properties of Group IV B Metallocene Polyoximes. Org. Coat. Plast. Chem..

[23] Carraher C., Linville R., Manek T., Blaxall H., Taylor J.R., Torre L. (1981). Electrical Properties of Group IV B Metallocene Polyoximes. Electrical Properties of Polymers.

[24] Battin A., Carraher C. (2008). Effect of doping by exposure to iodine vapor on the electrical conductivity of the polyamine from titanocene dichloride and 2-nitro-p-phenylenediamine. J. Polym. Mater..

[25] Carraher C. (1971). Synthesis of titanium polyesters. J. Polym. Sci..

[26] Carraher C. (2005). Condensation metallocene polymers. J. Inorg. Organomet. Polym. Mater..

[27] Carraher C. (1971). Fiber forming and thermal properties of polyesters of group IV metals. Chem. Tech..

[28] Stewart H., Soldani W., Carraher C., Reckleben L. (1991). Polymeric auxin plant growth hormones based on the condensation products of indole-3-butyric acid with bis(cyclopentadieneyl)titanium IV dichloride and dipyridine manganese II dichloride. Inorganic and Metal-Containing Polymeric Materials.

[29] Carraher C., Carraher S., Stewart H. (2010). Metal-containing polymer structures for enhanced seed germination and plant growth. Adv. Environ. Bio..

[30] Carraher C., Foster V., Linville R., Stevison D., Venkatachalam R. (1988). Organotin and organotitanium-containing polydyes for color permanence, reduction of laser damage and biological resistance to rot and mildew. Adhesives, Sealants, and Coatings for Space and Harsh Environments.

[31] Roner M., Carraher C., Shahi K., Ashida Y., Barot G. (2009). Ability of group IVB metallocene polyethers containing dienestrol to arrest the growth of selected cancer cell lines. BMC Caner.

[32] Carraher C., Roner M., Shahi K., Ashida Y., Barot G. (2007). Synthesis, structural characterization, and anti-cancer evaluation of group IVB-metallocene polyethers containing the synthetic estrogen diethylstibestrol. J. Polym. Mater..

[33] Carraher C., Lessek P. (1972). Synthesis of titanium polyamines via the interfacial and aqueous solution techniques. Eur. Polym. J..

[34] Carraher C., Jorgensen S. (1978). Study of Associated Reaction Variables in the Synthesis of Titanium (IV) Polyamines and a Comparison of Synthesis by Different Techniques. J. Polym. Sci..

[35] Inzelt G. (2008). Conducting Polymers.

[36] Bott D. (1985). Electrically conducting polymers. Phys. Technol..

[37] Harum M., Saion E., Kassim A., Yahya N., Mahmud E. (2007). Conjugated conducting polymers: A brief overview. JASA.

[38] Kumar D., Sharma R.C. (1998). Advances in conductive polymers. Eur. Polym. J..

[39] Chan W.K. (2007). Metal containing polymers with heterocyclic rigid main chains. Coord. Chem. Revs..

[40] Barford W. (2009). Electronic and Optical Properties of Conjugated Polymers.

[41] Chaing C.K. (2003). The bromine doping of polyacetlyene. Physica A.

[42] Chaing C.K., Park Y., Heeger A., Shirakawa H., Louis E., MacDiarmid A. (1978). Conducting polymers: Halogen-doped polyacetlyene. J. Chem. Phys..

[43] Bekkali A., Thurzo I., Kampen T., Zahn D., Dietrich R. (2004). Impedance spectroscopy study of metal-organic-metal structures. Appl. Surf. Sci..

[44] Cotton F.A., Wilkerson G. (1988). Advanced Inorganic Chemistry.

[45] Silverstein R., Webster F., Kiemle D. (2005). Spectrometric identification of organic compounds.

[46] Rao C.N.R. (1963). Chemical Application of Infrared Spectroscopy.

